# Maccabi-RED, mHealth innovation in community emergency care: a 4-year analysis of adoption patterns and impact on healthcare utilization

**DOI:** 10.3389/fdgth.2026.1752557

**Published:** 2026-04-13

**Authors:** Osnat Bashkin, Tamar Shalom, Limor Adler, Ilan Yehoshua

**Affiliations:** 1 Department of Public Health, Ashkelon Academic College, Ashkelon, Israel; 2 Department of Health Systems Management, The College of Law and Business, Ramat Gan, Israel; 3 Health Division, Maccabi Health Services, Tel Aviv, Israel; 4 Department of Family Medicine, Faculty of Medicine and Health Sciences, Tel Aviv University, Tel Aviv, Israel

**Keywords:** community-based care, digital health, emergency department overcrowding, emergency medical services, healthcare utilization, mobile health, patient technology adoption, technology acceptance

## Abstract

**Introduction:**

Emergency department overcrowding due to non-urgent visits places a considerable burden on the healthcare system. Mobile health (mHealth) technologies offer potential solutions by providing community-based alternatives for emergency care.

**Methods:**

In this study, we analyzed 4 years of implementation data from Maccabi-RED, a smartphone app-based emergency care service launched in 2019 by Israel's second-largest healthcare maintenance organization. We retrospectively analyzed electronic health records for all patient-initiated emergency care requests made through the Maccabi-RED app between January 2020 and December 2023. Our analysis encompassed 48,972 approved requests from 41,313 patients, comprising demographic characteristics, appointment adherence patterns, and subsequent healthcare utilization within 7 days. Statistical analyses included descriptive statistics, chi-square tests, t-tests, and multivariable logistic regression models.

**Results:**

Overall appointment attendance was 76.9%, improving from 52.4% in 2020 to 80.0% in 2023. Older patients (>51 years) had 29% higher attendance odds compared with younger patients (<19 years). Foreign body emergencies showed the highest attendance rates (72.6%), while surgical cases had the lowest (17.2%). The median wait time from request to appointment was 30.5 min, decreasing from 44.0 min in 2020 to 30.0 min in 2021–2023. Patients attending Maccabi-RED appointments had 16% lower odds of subsequent family physician visits and 41% lower odds of emergency medical center visits within 7 days, with no increase in emergency department visits or hospitalizations. However, geographic disparities emerged, with residents in peripheral areas showing lower attendance rates despite shorter wait times.

**Discussion:**

This study demonstrates that smartphone-based emergency care services can effectively reduce the burden on the healthcare system while maintaining patient safety, although targeted interventions are needed to address geographic and demographic disparities in access and utilization.

## 1 Introduction

Emergency medical services (EMS) are a vital component of the healthcare system. Often, patients arrive at hospital emergency departments (EDs) with non-urgent problems due to a lack of other treatment options in the community, which increases staff workload and imposes an economic burden on healthcare systems (1). Because EDs are constantly under pressure due to limited capacity, understaffing, and resource limitations (2), patient encounters with the medical service may be adversely affected (3).

The patient experience in EDs is determined by several factors, including interpersonal communication, information transfer, patient expectations, recognition of emotional needs, and actual wait times (4). The overcrowded environment of EDs, exacerbated by non-urgent visits, negatively impacts the quality of care and patient safety and may lead to long waiting times and diagnostic and treatment delays, which ultimately affect both care costs and the patient experience (5). In addition, highly crowded EDs expose medical staff to burnout and increased stress, which can decrease productivity (6, 7). To manage the existing load in EDs and improve the service provided to patients, various models for delivering EMS in the community have been developed (8), including emergency walk-in clinics or community health centers and community paramedic services (9, 10).

Connected health technologies are transforming healthcare delivery by enabling seamless integration among patients, providers, and health systems through digital platforms (11). The paradigm shift toward patient-centered, technology-mediated care has created diverse opportunities in EMS to address the complex challenges of providing high-quality and safe health services through innovative socio-technical models (2, 12).

Mobile digital medicine (mHealth) is expected to reduce costs, increase accessibility, streamline processes, and diminish inequality. Moreover, mHealth shows positive effects in providing EMS. For example, a recent meta-analysis systematically reviewed the technical features of existing mobile apps and evaluated their impact on the patient outcomes of out-of-hospital cardiac arrest under various emergency response strategies. The analysis of 13 mobile apps revealed that, compared to traditional EMS, mobile apps significantly improved bystander interventions and patient outcomes, such as survival to discharge, 30-day survival, and return of spontaneous circulation upon hospital admission (13). A study conducted in Thailand found that using an app for triage by patients and medical staff may be useful in classifying symptoms and determining patients' medical status before they visit the ED, thereby improving treatment effectiveness (14).

Despite the increased development of innovative digital health apps, one of the main challenges facing mHealth is the adoption and acceptance of new health technologies by users, as well as ensuring their continued use over time. A recent study on mobile digital health for EMS examined the usability, effectiveness, and user satisfaction of a mobile digital health app that was designed to guide parents through a decision tree combining symptoms and medical advice to determine whether their children's symptoms can be treated at home, whether a physician should be seen, or whether it is necessary to seek emergency medical care (15). When users rated the app's usability as good to excellent, it significantly influenced their decision to adopt and use the app again.

In Israel, the provision of mobile digital health services by healthcare maintenance organizations (HMOs) offers an opportunity to study their usefulness in a population with one of the highest mobile phone usage rates in the world and a high acceptance rate of technological innovations. A recent study that examined the characteristics of mHealth use among an adult population in Israel reported usage rates of mobile health apps of approximately 61%. Of these, 81% reported using apps provided by health funds. Moreover, half of the study participants believed that using mHealth apps could help them to discuss health issues with medical professionals (16).

Maccabi-RED was developed and launched in 2019 by Maccabi Healthcare Services, which is Israel's second-largest HMO, providing medical care for over 2.6 million people. This service was designed to provide emergency care in community settings, reducing the need for patients to visit the ED. In an emergency, patients can activate the Maccabi-RED service directly from their smartphone app to request an urgent visit, also known as a RED visit. In the next step, the patient can select from a preset list of emergencies (for instance, “foreign body in the eye” or “limb laceration”). If required, the App requests more information from the patient (time of occurrence and specific area in the body, duration of symptoms, intensity of symptoms, location of incision, amount of bleeding), particularly to characterize the situation and its urgency. An instant push notification is sent through the unified electronic health record (EHR) system to all relevant physicians' computers (working in clinics within a predefined distance from the patient's location), allowing them to accept or reject the request for a RED visit. When a physician accepts the request, the patient is directed to the physician's clinic, and other physicians no longer see the notification. Physicians participating in the Maccabi-RED network are all trained, preregistered, and preapproved by a medical supervisor. Alternatively, any physician or nurse can activate the service on behalf of the patient (by contacting a dedicated phone number or by using the patient's EHR) (17).

Such a service could dramatically reduce ED visits and hospitalization days, cut patient transit and waiting times, improve quality of care, and increase patient satisfaction. In addition, healthcare systems could benefit from significantly reduced costs. However, despite the proliferation of mHealth, few studies have examined its real-world implementation and effectiveness in emergency care contexts. Moreover, there is limited empirical evidence on comprehensive community-based digital EMS that integrate patient-initiated requests with physician networks. The lack of longitudinal data on digital EMS particularly limits understanding of how these platforms evolve over time and whether they achieve their intended goals of reducing ED burden. The Technology Acceptance Model suggests that perceived usefulness and ease of use influence technology adoption (18, 19), while the Unified Theory of Acceptance and Use of Technology emphasizes the role of facilitating conditions and social influence in healthcare technology uptake (20). Understanding these factors is crucial for evaluating digital EMS such as Maccabi-RED.

In the present study, we quantified and characterized the use of Maccabi-RED by patients during 4 years of operation and assessed its effects on ED burden. Specifically, we addressed three key research questions: [1] What are the demographic and clinical characteristics of patients who use mobile emergency care services? [2] What factors influence patient adherence to scheduled emergency appointments requested via the Maccabi-RED app? [3] How does mobile emergency care affect subsequent healthcare utilization patterns?

## 2 Methods

### 2.1 Data and study setting

Data for this retrospective study was obtained from the EHRs of Maccabi Healthcare Services. We analyzed all requests for emergency care made by patients through the Maccabi-RED app between January 2020 and December 2023 that were approved and that received an appointment for emergency care in a community clinic (*N* = 48,972 approved app requests). In addition, data from Maccabi-RED visits in community clinics, made at the patients' request through the application, were included in the study (*N* = 37,659 visits). Emergency visits to community clinics referred by a family physician or nurse to the Maccabi-RED service but not initiated by patients through the Maccabi-RED app were excluded from the study.

### 2.2 Ethical considerations

This study was approved by the institutional review board of Maccabi Healthcare Services (0100-23-MHS). As a retrospective medical record review of de-identified patient data, no informed consent was required.

### 2.3 Study population

The dataset included all patients registered with Maccabi Healthcare Services living in Israel who used the Maccabi-RED app to request at least one scheduled appointment for emergency care at a community clinic during the study period (January 2020 to December 2023) (*N* = 41,313 patients). The data represented a geographically and demographically diverse population of Israel.

### 2.4 Variables

For each approved Maccabi-RED request, we extracted the following demographic characteristics (Table 1): age, sex, socioeconomic status, sector (general population, orthodox Jewish, or Arab), residency (urban vs. periphery), smoking status, and comorbidities. We also retrieved data on the type of emergency care requested (orthopedics, foreign body, surgery, obstetrics, and gynecology) and the time from the Maccabi-RED service request in the app to the visit.

**Table 1:** Demographic and clinical characteristics of Maccabi-RED users by appointment attendance status (*N* = 48,972).

Variables	Approved Maccabi-RED requests with scheduled appointments *N* = 48,972 (100%)	No-show Maccabi-RED scheduled appointments *N* = 11,313 (23.1%)	Maccabi-RED visits *N* = 37,659 (76.9%)	*p*-value
Year, *n* (%)				
2020	3,876 (7.9)	1,845 (16.3)	2,031 (5.4)	<0.001
2021	9,079 (18.5)	2,200 (19.4)	6,879 (18.3)	
2022	16,965 (34.6)	3,450 (30.5)	13,515 (35.9)	
2023	19,052 (38.9)	3,818 (33.7)	15,234 (40.5)	
Age in years at index date, mean ± SD and median [IQR]	37.3 ± 22.0	33.8 ± 20.0	38.4 ± 22.4	0.009
	36.0 [21.0–54.0]	32.0 [18.0–48.0]	38.0 [22.0–56.0]	
Age group, *n* (%)				
<19 years	11,427 (23.3)	2,833 (25.0)	8,594 (22.8)	<0.001
19–33 years	11,118 (22.7)	3,109 (27.5)	8,009 (21.3)	
34–50 years	11,714 (23.9)	2,919 (25.8)	8,795 (23.4)	
>51 years	14,713 (30.0)	2,452 (21.7)	12,261 (32.6)	
Area of residence, *n* (%)				
Periphery	2,063 (4.2)	651 (5.8)	1,412 (3.7)	<0.001
Center	46,909 (95.8)	10,662 (94.2)	36,247 (96.3)	
Ethnicity, *n* (%)				
Arab	1,141 (2.3)	288 (2.6)	853 (2.3)	0.175
Orthodox Jewish	1,690 (3.5)	397 (3.5)	1,293 (3.5)	
General	45,811 (94.2)	10,517 (93.9)	35,294 (94.3)	
Sex, *n* (%)				
Male	21,466 (43.8)	4,926 (43.5)	16,540 (43.9)	0.478
Female	27,506 (56.2)	6,387 (56.5)	21,119 (56.1)	
Socioeconomic status, *n* (%)				
Low	5,425 (11.2)	1,143 (10.2)	4,282 (11.5)	<0.001
Middle	24,043 (49.5)	5,204 (46.5)	18,839 (50.4)	
High	19,093 (39.3)	4,842 (43.3)	14,251 (38.1)	
Smoker, *n* (%)	9,702 (19.8)	2,228 (19.7)	7,474 (19.8)	0.721
Comorbidities, *n* (%)				
Oncologic disease	3,725 (7.6)	664 (5.8)	3,057 (8.1)	<0.001
Cognitive impairment	622 (1.3)	90 (0.8)	532 (1.4)	<0.001
Homecare	38 (0.1)	11 (0.1)	27 (0.1)	<0.001
Prediabetes	11,576 (23.6)	2,145 (19.0)	9,431 (25.0)	<0.001
Hypertension	6,960 (14.2)	1,096 (9.7)	5,864 (15.6)	<0.001
Cardiovascular disease	3,405 (7.0)	524 (4.6)	2,881 (7.7)	<0.001
Immunocompromised	1,176 (2.4)	202 (1.8)	974 (2.6)	<0.001
Inflammatory bowel disease	599 (1.2)	131 (1.2)	468 (1.2)	0.472
Chronic respiratory disease	830 (1.7)	128 (1.1)	702 (1.9)	<0.001
Chronic renal disease	1,795 (3.7)	278 (2.5)	1,517 (4.0)	<0.001
Diabetes	3,490 (7.1)	576 (5.1)	2,914 (7.7)	<0.001
Fall risk	4,616 (9.4)	595 (5.3)	4,021 (10.7)	<0.001
Hypercoagulability	117 (0.2)	27 (0.2)	90 (0.2)	0.995
Bone disease	3,340 (6.8)	496 (4.4)	2,844 (7.6)	<0.001
Emergency care specialty, *n* (%)				
Orthopedics	9,791 (20.0)	2,821 (24.9)	6,970 (18.5)	<0.001
Foreign body	33,159 (67.7)	5,817 (51.4)	27,342 (72.6)	
Surgery	3,277 (6.7)	1,942 (17.2)	1,335 (3.5)	
Obstetrics and Gynecology	2,745 (5.6)	733 (6.5)	2,012 (5.3)	

Primary outcome variables included: [1] appointment adherence, calculated as the percentage of patients attending scheduled appointments within the specified time window; and [2] healthcare resource utilization, measured by subsequent family/pediatric physician visits, community emergency center visits, ED admissions, and hospitalizations, within 7 days of the visit (for any cause).

### 2.5 Data analysis

Statistical analysis included descriptive statistical analyses of all patient demographics and study variables. Results are presented as distributions for all categorical variables and measures of central tendency for continuous variables (patient age and time from the Maccabi-RED request to the visit).

We compared the characteristics of Maccabi-RED patients who attended their scheduled appointment to those of Maccabi-RED patients who did not attend the scheduled appointment using a chi-square test for categorical variables and a two-sided *t*-test for continuous variables. The Mann–Whitney test and Kruskal–Wallis test were used to examine differences in times from the Maccabi-RED app activation to the visit. Finally, logistic regression models were used to assess patient attendance at scheduled appointments by demographic variables and to evaluate healthcare resource utilization by patient attendance at scheduled appointments. Missing data were categorized as “unknown” and were not accounted for in any analysis. Data was analyzed using IBM SPSS Statistics 29.0 software. Statistical significance was determined at *p* < 0.05.

## 3 Results

A total of 94,795 Maccabi-RED app requests for emergency care were recorded during the study period by 77,508 different patients. Of these, 48,972 Maccabi-RED requests (51.6%) were approved and received an emergency care appointment at a community clinic. Table 1 presents the demographic and clinical characteristics of patients with approved Maccabi-RED requests, stratified by appointment attendance. Most requests (76.9%) resulted in completed visits. Patients who attended their appointments were significantly older (mean age, 38.4 vs. 33.8 years; *p* < 0.001) and had higher rates of chronic comorbidities, including prediabetes (25.0% vs. 19.0%), hypertension (15.6% vs. 9.7%), and cardiovascular disease (7.7% vs. 4.6%). The geographic distribution showed that patients living in peripheral areas had lower attendance rates (3.7% vs. 5.8% no-shows, *p* < 0.001). Foreign body-related emergencies comprised the largest category (67.7% of all approved requests), with higher attendance rates compared to surgical emergencies.

Table 2 presents the results of a multivariable logistic regression analysis for factors associated with appointment attendance. After adjusting for all variables, several factors emerged as significant predictors. Patients in later study years showed progressively higher odds of attendance compared to 2020, suggesting improved service adoption over time. Older adults (>51 years) had a 29% higher attendance rate than young people (<19 years). The type of emergency was strongly associated with attendance, with foreign body cases showing 76% higher odds of attendance compared to orthopedic emergencies, while surgical cases had 71% lower odds of appointment attendance. Further analysis revealed that 14.1% (*n* = 8,742) of patients used the Maccabi-RED app more than once during the study period. While 85.2% of repeat users attended the scheduled appointment, 75.1% of patients who made only one request through the app attended the scheduled appointment, likely reflecting increased adherence among repeat users.

**Table 2:** Multivariable logistic regression analysis of factors associated with Maccabi-RED appointment attendance.

Variables	OR [95% CI]
Index year	
2020	Ref. (1.00)
2021	2.61 [2.40–2.84]
2022	3.09 [2.86–3.34]
2023	3.09 [2.86–3.35]
Age group, years	
<19	Ref. (1.00)
19–33	0.95 [0.89–1.01]
34–50	0.98 [0.92–1.05]
>51	1.29 [1.18–1.40]
Peripheral residence	0.83 [0.74–0.93]
Ethnicity	
Arab	0.87 [0.75–1.01]
Orthodox Jewish	0.95 [0.83–1.09]
General	Ref. (1.00)
Sex (male vs. female)	0.88 [0.84–0.92]
Socioeconomic status	
Low	Ref. (1.00)
Middle	0.86 [0.79–0.94]
High	0.77 [0.70–0.84]
Smoker	0.99 [0.93–1.04]
Comorbidities	
Oncologic disease	0.95 [0.85–1.06]
Cognitive impairment	0.95 [0.75–1.21]
Homecare	0.51 [0.23–1.11]
Prediabetes	1.12 [1.05–1.19]
Hypertension	1.03 [0.94–1.13]
Cardiovascular disease	1.16 [1.04–1.30]
Immunocompromised	1.17 [0.99–1.39]
Inflammatory bowel disease	0.97 [0.79–1.20]
Chronic respiratory disease	0.92 [0.75–1.12]
Chronic renal disease	0.97 [0.84–1.13]
Diabetes	1.04 [0.92–1.16]
Fall risk	1.27 [1.12–1.44]
Hypercoagulability	0.62 [0.39–0.98]
Bone disease	1.08 [0.96–1.21]
Emergency care specialty	
Orthopedics	Ref. (1.00)
Foreign body	1.76 [1.66–1.86]
Surgery	0.29 [0.27–0.32]
Obstetrics and Gynecology	1.43 [1.27–1.61]

CI, confidence interval.

The time from activating the Maccabi-RED app to the visit was calculated according to demographic variables. Analyses were based on attended visits only (*N* = 37,659) as time was not recorded for no-show cases. Table 3 presents the mean and median times from app activation to appointment across demographic and clinical subgroups. The median overall time was 30.5 min [interquartile range (IQR), 11.0–58.0]. Analysis showed a significant improvement in service efficiency over the study period (*p* < 0.001), with median wait times decreasing from 44.0 min in 2020 to 30.0 min in 2021–2023, suggesting system optimization and increased physician network capacity as the service matured. Demographic variations revealed notable disparities in access times. Patients in peripheral areas experienced shorter times (median, 22.0 vs. 31.0 min; *p* < 0.001), likely reflecting lower service demand in these regions. Arab patients had the shortest median times (21.0 min), while the general population had the longest (31.0 min). Emergency type significantly influenced wait times, with obstetrics/gynecology cases having the shortest delays (median, 2.0 min) and surgical cases having the longest (median, 34.0 min).

**Table 3:** Time from service request to appointment by patient demographic and emergency type.

Variables	Time from activating the Maccabi-RED app to visit* (minutes) mean ± SD median [IQR]	*p*-value^a^	Effect size
Index year			
2020 (*n* = 1,981)	52.7 ± 47.3 44.0 [23.0–68.0]	<0.001	0.01
2021 (*n* = 6,758)	40.2 ± 41.3 30.0 [11.0–56.0]		
2022 (*n* = 13,322)	39.4 ± 40.5 29.0 [11.0–56.0]		
2023 (*n* = 15,016)	41.5 ± 43.7 30.0 [10.0–59.0]		
Age at index date, years			
<19 (*n* = 8,479)	41.8 ± 40.5 31.0 [13.0–58.0]	<0.001	0.00
19–33 (*n* = 7,880)	42.6 ± 45.3 32.0 [9.0–61.0]		
34–50 (*n* = 8,635)	41.7 ± 43.2 31.0 [10.0–60.0]		
>51 (*n* = 12,083)	39.2 ± 41.3 28.0 [10.0–54.0]		
Socioeconomic status			
Low (*n* = 4,222)	36.3 ± 40.2 25.0 [8.0–50.0]	<0.001	0.00
Middle (*n* = 18,485)	41.7 ± 42.2 31.0 [12.0–58.0]		
High (*n* = 14,090)	41.9 ± 43.3 31.0 [11.0–60.0]		
Peripheral residence			
No (*n* = 35,679)	41.4 ± 42.5 31.0 [11.0–58.0]	<0.001	0.04
Yes (*n* = 1,398)	34.4 ± 40.6 22.0 [8.0–46.0]		
Ethnicity			
Arab (*n* = 849)	34.9 ± 46.8 21.0 [5.0–48.0]	<0.001	0.00
Orthodox Jewish (*n* = 1,286)	39.1 ± 43.0 26.0 [10.0–52.0]		
General (*n* = 34,730)	41.3 ± 42.4 31.0 [11.0–58.0]		
Emergency care specialty			
Orthopedics (*n* = 6,904)	39.3 ± 38.0 30.0 [12.0–56.0]	<0.001	0.03
Foreign body (*n* = 26,869)	42.6 ± 42.7 32.0 [12.0–60.0]		
Surgery (*n* = 1,317)	45.1 ± 50.1 34.0 [13.0–61.5]		
Obstetrics and gynecology (*n* = 1,987)	24.2 ± 45.3 2.0 [0.0–34.0]		

* Analyses are based on attended visits only (*N* = 37,659).

^a^ Mann–Whitney test or Kruskal–Wallis test.

Table 4 examines healthcare utilization outcomes within 7 days of the Maccabi-RED request, comparing patients who attended their appointments with those who did not. After adjustment for demographic and clinical factors, patients who attended Maccabi-RED visits had 16% lower odds of subsequent family physician visits and 41% lower odds of emergency medical center visits. No significant differences were observed in ED visits or hospitalizations, suggesting that Maccabi-RED effectively addresses non-life-threatening emergency needs while maintaining patient safety.

**Table 4:** Healthcare utilization outcomes within 7 days following Maccabi-RED requests by appointment attendance status.

Outcome noted within 7 days of the Maccabi-RED request	No/Yes	No-show Maccabi-RED scheduled appointments *N* = 11,313	Maccabi-RED visits *N* = 37,659	Crude OR [95% CI]	Adjusted OR [95% CI]^a^
Family physician/pediatrician visit	No	6,416 (56.7)	23,834 (63.3)	0.76 [0.73–0.79]	0.84 [0.80–0.88]
	Yes	4,897 (43.3)	13,825 (36.7)		
ED visit	No	11,285 (99.8)	37,584 (99.8)	0.80 [0.52–1.34]	0.82 [0.52–1.30]
	Yes	28 (0.2)	75 (0.2)		
Hospitalization	No	11,298 (99.9)	37,604 (99.9)	1.10 [0.62–1.95]	1.02 [0.55–1.86]
	Yes	15 (0.1)	55 (0.1)		
Emergency medical center visit	No	11,090 (98.0)	37,253 (98.9)	0.54 [0.46–0.64]	0.59 [0.49–0.70]
	Yes	223 (2.0)	406 (1.1)		

CI, confidence interval.

^a^ Adjusted for all variables included in the logistic regression in Table 2.

## 4 Discussion

This 4-year analysis of the Maccabi-RED digital EMS provides valuable insights into the implementation of mHealth for enhancing community-based emergency care. The results demonstrate substantial growth in the adoption of mHealth emergency services, with attendance rates improving from 52.4% in 2020 to 80.0% in 2023, while revealing significant disparities in access and utilization patterns across different population groups. The number of visits indicates strong patient support for community-based alternatives to ED visits. Furthermore, the increase in attendance among repeat users of the app may suggest growing trust among users.

### 4.1 Geographical disparities

Patients living in peripheral areas had lower attendance rates (3.7% vs. 5.8% no-shows) but shorter wait times (22.0 vs. 31.0 min), revealing important geographic disparities in usage patterns. This finding has direct practical implications for healthcare policy, suggesting that wait time alone is not the primary barrier to service utilization in these populations, and that targeted outreach and digital literacy interventions are needed. This finding aligns with previous studies showing that rural residents and patients residing farther from medical centers use patient portals, EHRs, and telehealth services less than urban populations, even when device ownership is comparable (21, 22). Populations in rural or remote areas tend to face more substantial obstacles to e-healthcare (23). Consequently, they may encounter greater barriers to service attendance, including transportation challenges, work-related constraints, and distinct healthcare-seeking behaviors. A systematic review found that information and communication technology infrastructure, trained personnel, illiteracy, and the lack of multilingual text and voice messages were major challenges hindering the effective usage of mHealth technologies in sub-Saharan African countries (24). The shorter wait times in peripheral areas likely reflect higher Maccabi-RED physician availability due to reduced service demand in those areas. However, the higher no-show rates among peripheral residents may indicate that digital emergency services, while improving access, do not fully address the complex barriers faced by geographically distant populations. In addition, the overall low representation of minority populations in the service (Arab, 2.3%; Orthodox Jewish, 3.5%) suggests potential barriers to adoption that warrant further investigation. Previous research on mHealth adoption in Israel found that, while overall usage rates are high (61%), with 81% using health HMO-provided apps (16), disparities in digital health literacy and technology acceptance may affect minority communities differently. According to the Technology Acceptance Model, perceived usefulness and ease of use influence adoption, while cultural factors and language barriers impact the engagement of minority populations with mHealth services (19). System-level interventions can bridge these gaps by proactively reaching high-disparity communities, facilitating access, and promoting perceptions of the value of mHealth solutions (25).

### 4.2 Impact on healthcare utilization outcomes

The most clinically significant finding relates to the impact of service attendance on subsequent healthcare utilization. Patients who attended Maccabi-RED appointments had 16% lower odds of subsequent family physician visits and 41% lower odds of emergency medical center visits within 7 days. These findings demonstrate that this mHealth tool, which provides community-based emergency care services, can effectively reduce the burden on the healthcare system while maintaining high-quality care. Emergency medical center visits represent a substantial cost and capacity burden on the healthcare system. Even a modest reduction in such visits at the population level translates into meaningful cost savings and capacity gains for the healthcare system. The substantial reduction in emergency medical center visits particularly supports the intended goal of the service of diverting non-life-threatening cases from higher-acuity care settings. This pattern aligns with previous research showing that mobile health interventions in emergency care can reduce inappropriate emergency department utilization while maintaining patient safety (14). The Houston Fire Department initiated the Emergency Telehealth and Navigation (ETHAN) program in 2014, which combines telehealth, social services, and alternative transportation to divert primary care-related patients away from EDs when possible (26). This mobile technology-driven delivery model effectively reduced unnecessary ED ambulance transport to urban EDs by 56% and increased EMS unit productivity. In another evaluation of a mobile crisis service intervention implemented in the USA, researchers reported that youths who received mobile crisis services had a significant reduction in the odds of subsequent health ED visits compared with youths in the comparison sample (27). Our findings suggest that an mHealth tool supporting community EMS can serve as an effective intermediary care model, appropriately triage patients, and reduce the unnecessary utilization of higher-acuity services. The maintained safety profile, evidenced by no increase in ED visits or hospitalizations, indicates that the service successfully identifies and manages cases appropriate for community-based care.

### 4.3 Emergency type variation and service optimization

The dominance of foreign body removal cases (67.7% of requests) with high attendance rates (72.6%) vs. the low attendance rates for surgical emergencies (17.2% no-shows) highlights important service design considerations. The dramatic difference in times between obstetrics/gynecology cases (2.0 min) and surgical cases (34.0 min) suggests that the service functions optimally for specific types of emergencies but may require optimization for others. The high no-show rates for surgical emergencies may reflect the complexity of these cases, patient anxiety about procedures, or inadequate preparation time. This finding suggests that different emergency types may require tailored approaches to ensure patient engagement and facilitate appointment management. In the context of community-based emergency care, a median wait time of 30.5 min is practically meaningful when compared to typical ED wait times in Israel and internationally, which frequently range from 2 to 6 h (28). This difference represents a substantial improvement in patient experience and time efficiency. Given the large sample size of this study, it is important to interpret statistically significant findings in light of their practical and clinical relevance. While the reduction in median service time from 44.0 in 2020 to 30.0 min in 2023 is statistically significant, its administrative importance lies primarily in what it reflects, namely, the growth of the Maccabi-RED physician network and improved system capacity over time. More clinically impactful are the substantial reductions in emergency medical center utilization (41% lower odds) and the marked improvement in attendance rates (52.4% to 80.0%), which together provide strong evidence for the real-world effectiveness of this mHealth service.

### 4.4 Limitations and future directions

Several limitations should be considered when interpreting these findings. First, we excluded physician and nurse-initiated Maccabi-RED visits, which may introduce selection bias toward more motivated or technologically skilled patients. Second, while the rate of subsequent ED visits and hospitalizations was low and did not differ significantly between attendees and non-attendees, we cannot fully exclude the possibility that a subset of these cases represented triage failures associated with clinical worsening. Future studies should incorporate clinical severity data, ED triage classifications, and downstream outcome measures such as length of stay and intensive care unit admission rates to more comprehensively evaluate the safety profile of community-based digital emergency triage systems. Such analyses would provide stronger evidence regarding the appropriateness of case selection and the clinical consequences of any potential triage errors. Third, while our data captures some downstream healthcare utilization within 7 days, we cannot determine whether no-show patients sought care outside the Maccabi network, resolved their emergency without further intervention, or experienced clinical deterioration beyond our follow-up window. Future research should incorporate mixed methods approaches, combining electronic health record linkage across healthcare providers with direct patient surveys or interviews, to comprehensively characterize the experiences and outcomes of patients who do not attend digital emergency care appointments. Finally, we did not assess physicians' and healthcare staff's experiences with the Maccabi-RED service, including workflow impacts and professional satisfaction. Future research should examine longer-term outcomes, explore barriers to service adoption among minority populations in depth, and investigate strategies to improve adherence rates for complex emergency types. In addition, cost-effectiveness analyses would provide valuable insights for healthcare policy decisions regarding the implementation of mHealth emergency care tools.

### 4.5 Conclusions and implications

This comprehensive 4-year analysis provides robust evidence for the clinical effectiveness and growing acceptance of digital mobile emergency care services while revealing critical implementation challenges that must be addressed for successful mHealth integration. The progressive improvement in attendance rates over the study period suggests that digital health services require time for community adoption and optimization, demonstrating the importance of allowing connected health platforms to mature within local healthcare ecosystems. The Unified Theory of Acceptance and Use of Technology model emphasizes the role of facilitating conditions and social influence in technology uptake, which may explain the observed improvement as the service became more established and accepted within the community.

The substantial reduction in emergency medical center visits among Maccabi-RED app users demonstrates the potential of digital mobile emergency care to address healthcare system capacity challenges while improving patient access to appropriate care. However, the geographic and demographic disparities identified highlight the need for targeted interventions to ensure equitable access to digital health innovations.

This study provides real-world evidence supporting the effectiveness of mHealth in emergency care services while identifying important areas for service improvement and equity considerations in digital health implementation.

## Data Availability

The datasets presented in this article are not readily available because according to the Israel Ministry of Health regulations, individual-level data cannot be shared openly. Specific requests for remote access to de-identified community-level data should be referred to the Kahn Sagol Maccabi Research and Innovation Center, Maccabi Healthcare Services. Requests to access the datasets should be directed to yehoshua_i@mac.org.il.
